# Examining the Prediction of COVID-19 Contact-Tracing App Adoption Using an Integrated Model and Hybrid Approach Analysis

**DOI:** 10.3389/fpubh.2022.847184

**Published:** 2022-05-24

**Authors:** Ali Alkhalifah, Umar Ali Bukar

**Affiliations:** ^1^Department of Information Technology, College of Computer, Qassim University, Buraidah, Saudi Arabia; ^2^Department of Mathematical Sciences, Computer Science Unit, Taraba State University, Jalingo, Nigeria

**Keywords:** COVID-19, contact-tracing app, health, privacy, behavioral intention (BI), technology adoption

## Abstract

COVID-19 contact-tracing applications (CTAs) offer enormous potential to mitigate the surge of positive coronavirus cases, thus helping stakeholders to monitor high-risk areas. The Kingdom of Saudi Arabia (KSA) is among the countries that have developed a CTA known as the Tawakkalna application, to manage the spread of COVID-19. Thus, this study aimed to examine and predict the factors affecting the adoption of Tawakkalna CTA. An integrated model which comprises the technology acceptance model (TAM), privacy calculus theory (PCT), and task-technology fit (TTF) model was hypothesized. The model is used to understand better behavioral intention toward using the Tawakkalna mobile CTA. This study performed structural equation modeling (SEM) analysis as well as artificial neural network (ANN) analysis to validate the model, using survey data from 309 users of CTAs in the Kingdom of Saudi Arabia. The findings revealed that perceived ease of use and usefulness has positively and significantly impacted the behavioral intention of Tawakkalna mobile CTA. Similarly, task features and mobility positively and significantly influence task-technology fit, and significantly affect the behavioral intention of the CTA. However, the privacy risk, social concerns, and perceived benefits of social interaction are not significant factors. The findings provide adequate knowledge of the relative impact of key predictors of the behavioral intention of the Tawakkalna contact-tracing app.

## 1. Introduction

The usage of mobile contact-tracing apps (CTAs) has developed exponentially due to the severe impact of COVID-19. One major reason behind this development is as a result of the effort made to contain the spread of the coronavirus (1–3). Stakeholders are concerned about easing movement control and physical distancing (4) and the weaknesses of manual contact tracing (5). These circumstances have presented a compelling reason for stakeholders to depend on digital monitoring, enabling more effective, engaging, and nearly instant tracing of cases than is the case with traditional manual tracing methods (6, 7). The mobile contact-tracing apps are technology-based solutions used to help increase the conventional contact-tracing process. The apps operate by identifying contacts at risk of COVID-19 automatically (2). Hence, the contact-tracing apps exchange information of personal phones within close range, which informs people of infected individuals. Also, the apps serve as an anchor point to inform citizens and provide suggestions on whether they should go to isolation or not.

Many contact-tracing solutions have been introduced by various countries and technology companies (8, 9). Recent advancements by Google and Apple in these efforts are providing crisis managers with many features, such as personalized messages, depending on the mobile user's current geographic location (9). Additionally, mobile phones, due to their ease of use, are influencing citizens, with actual usage statistics demonstrating a consistent rise in adoption (10), indicating that many countries are shifting to technology-based platforms. As a result, several studies have investigated these applications that monitor infected individuals and their surroundings (10, 11). One of the primary issues is the personal information provided by users (2, 12). The use of mobile devices enables data capture that can be shared with third-party developers, analytics, and decision-makers (13). This may raise concerns about the user's privacy, which are even greater when the authorities or other public entities hold such sensitive information (14–16). Furthermore, the many regulations used to protect users' privacy have increased the importance of this issue (17, 18). Hence, all organizations and stakeholders must contribute to knowledge and enhanced understanding of the rights, perceptions, and behaviors of users of CTAs.

Some studies have reported that privacy is the primary concern for users of COVID-19 tracing apps (2, 10). This concern negatively influences the individual's intention to share or disclose personal information, therefore blocking the adoption of the app. Others have indicated that the potential benefits of these apps may outweigh the risks of being exposed to the coronavirus (19). However, contrary opinions have indicated that this is not simply the case of a pandemic, such as COVID-19, as individual social activities are threatened (4). Hence, essential elements influencing the acceptance of CTAs, especially the risk of losing social involvement, have not been well-examined. Moreover, in practice, the apps' effectiveness is frequently promoted as enabling immediate benefits to be obtained by users through factors such as the apps' usefulness. Still, most people hold some concerns about the apps' appropriateness and ease of use: as emphasized in the literature, although appealing, the apps are fraught with issues (10, 20).

Although, Kaspar (21) highlighted that most users are interested and eager to use these types of app, however, it is still vehemently clear that the factors that encourage users' intention to use and adopt these apps need to be researched. Accordingly, the Kingdom of Saudi Arabia (KSA), one of the many countries that have introduced a CTA (known as Tawakkalna), is using the app to manage the spread of COVID-19. Therefore, this study primary focus is to investigate the factors influencing users' willingness to utilize an app, which has the ability to provide users with information concerning potential association with individual's who may have contracted the virus. To achieve its aim, the study is guided by the following research question:

- RQ: To what extent does existing technology adoption factors influences the behavioral intention of Tawakkalna?

By applying the task-technology fit (TTF) model, the technology acceptance model (TAM), and privacy calculus theory (PCT), this study examines the impact of factors, such as perceived privacy risk, perceived social risk, and social interaction, as well as perceived ease of use (PEoU), perceived usefulness (PU), and TTF on behavioral intention of Tawakkalna. Also, this study investigate the predictive relevance of the key indicators *via* machine learning (ML) technique that has received little attention in the current literature (22, 23), particularly the application of artificial neural network (ANN). Moreover, this study is among the first to test the predictors of behavioral intention of CTA using an integrated model and a dual-stage SEM–ANN approach. Therefore, the following section summarizes the rationale and existing literature on the TTF model, the TAM, and PCT and presents a conceptual model of Tawakkalna acceptance, along with interrelated hypotheses. The research approach is then discussed. Next, the results section contains information on the hypotheses testing conducted. Finally, additional discussion and implications are offered to substantiate the conclusions, limitations, and recommendations for further research.

## 2. Literature Review

### 2.1. Background and Motivation

The speed at which COVID-19 cases are increasing and fear of overburdening health services has made numerous countries implemented measures such as lockdowns to restrict the virus spread (8). As a result, new technology-based strategies for identifying contacts have been suggested, mainly when case detection is aggressive (6, 7). The digital contact-tracing mobile apps have been developed by government and health authorities as a response to track association of individual and automatically provide instructions concerning self-isolation measures to potentially infected people. Also, Apple and Google have introduced a third-party apps on iOS and Android devices to facilitate the development of CTAs by the public health agencies worldwide (8, 9, 20). Although, Apple and Google assert that user privacy and security are central to the design, privacy concerns have been raised (20, 24).

The privacy concerns have caused difficulties with trust around users' consent and participation in downloading and using such apps. This is particularly true in liberal countries that support socially progressive opinions. Moreover, in those countries, the usage of such apps is optional, effectively negating their purpose. According to (25) as cited in (8), the effectiveness of contact-tracing apps depends on the number of people in the population who use them. Approximately 50–70% of the population within a region or a country is highly recommended. The scientific and epidemiological evidence suggests that CTAs can lower pandemic-related suffering and ease lockdowns (26). Interestingly, online surveys outcomes, conducted in advanced countries (France, United Kingdom, Italy, Germany, and the United States) has demonstrated strong support for the CTAs (11). However, as emphasized by (8), this does not imply that people will use the app.

Numerous studies indicate that CTAs can considerably contribute in limiting and halting the spread of COVID-19 by accelerating reporting and contact-tracing practices through enhanced proximity tracing, digital data flow, and geolocation monitoring (8, 27). They could play a critical role, given the widespread usage of internet-connected devices, increasing the speed with which many smartphone users can be monitored in real time to determine infection hotspots. Contact-tracing apps are critical to COVID-19 management measures in several countries (28). Additionally, they can be vital in flagging other illnesses, mainly when physical contact tracing is impossible. Indeed, work by Kucharski et al. (29) emphasized that combining testing and contact tracing significantly reduced the transmission of the coronavirus more than either self-isolation or mass testing.

**Figure 1:**
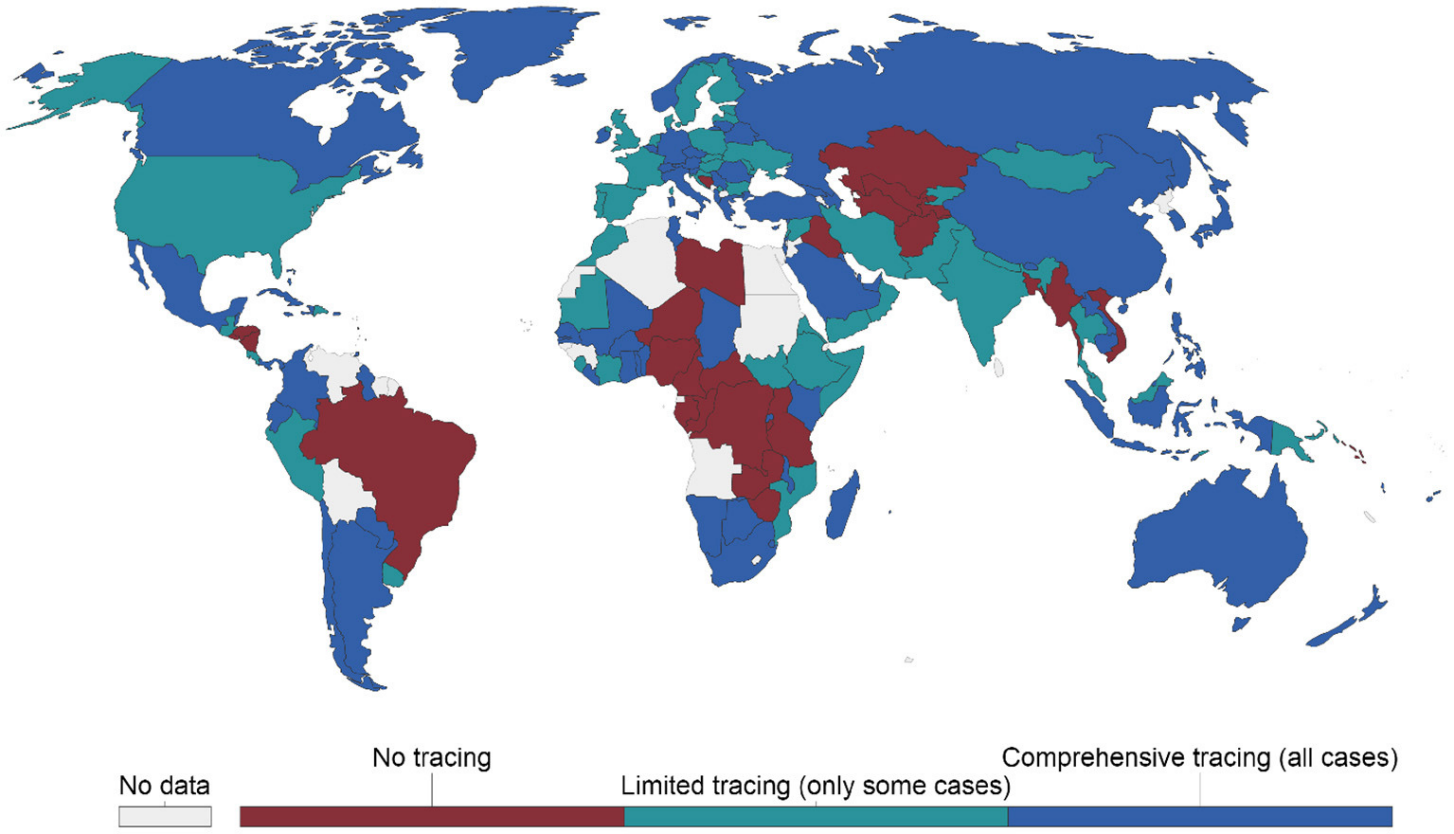
Countries with COVID-19 contact-tracing initiatives as of July 6, 2021. Source: Hale et al. (33).

Notably, the literature has expressed consensus regarding the difficulty of implementing contact tracing without infringing an individual's privacy (8, 9, 24). The threat to personal privacy was significant enough for Google and Apple to work on an exposure notification API, an application programming interface (API). The API enables public health organizations to deploy contact-tracing solutions designed to protect users' privacy and security. The efficiency of the apps in tracking and tracing individuals infected with COVID-19 has however been questioned in advanced countries by industry experts and academics. The apps have been limited by privacy, security, and technical issues, and their influence on the COVID-19 pandemic remains unknown (30, 31). Moreover, many countries worldwide have not made any effort to implement CTAs, and their COVID-19 management has been among the best (e.g., Mauritius, Tanzania, and Iceland) (32). Figure 1 presents countries across the world, showing those with no tracing, limited tracing, and comprehensive tracking.

#### 2.1.1. Adoption of CTA

The development of contact tracing apps and adoption is a never-ending problem for public health systems and policymakers (34). This study offers an evidence-based, situation-specific concern to understand better the theoretical and practical significance of CTA adoption in KSA. The literature review shows that most of the contact tracing apps adoption where conducted in liberal countries (10, 11, 34–41). According to the existing studies, the unified theory of acceptance and use of technology (UTAUT), privacy calculus theory (PCT), and extended technology adoption model (eTAM) dominated the studies of CTA adoption. Similarly, privacy concerns are the major issues studied in CTA adoption, and most of the literature shows that privacy is not a significant predictor of CTA. However, privacy concerns are not investigated in other regions, such as the Middle East and Africa. This calls for more studies to investigate this phenomenon in another geographical context.

Moreover, the current CTA adoption literature have integrated multiple theories in their studies. For example, work by Sharma et al. (36) integrated fairness theory (FT), dual calculus theory (DCT), protection motivation theory (PMT), theory of planned behavior (TPB), and Hofstede's cultural dimension theory (HCDT) to investigate the adoption intention of CTA. The literature review has identified a few studies that employed a two-stage analysis to investigate CTA adoption. Specifically, Duan and Deng (37) employed CB-SEM with an artificial neural network (ANN) approach to investigate the CTA adoption through the lens of the unified theory of acceptance and use of technology and privacy calculus theory in Australia. In contrast, the study by Nguyen et al. (39) investigated the extended technology adoption model through PLS-SEM and fuzzy set/qualitative comparative analysis (fsQCA). The summary of the review findings is presented in Table 1. This review provides a validated list of elements influencing people's CTA adoption concerns and attitudes in various countries. However, similar studies in the context of KSA are lacking to allow policymakers to take systematic steps to address these issues and enhance CTA acceptance.

**Table 1:** Summary of existing CTA adoption studies.

**References**	**Theory**	**Analysis method**	**Findings**	**Country**
Velicia-Martin et al. (10)	Extended TAM	Empirical, PLS-SEM	Usefulness and ease of use are significant predictors. However, there is no cause of privacy concern.	Spain
Altmann et al. (11)	NA	Empirical, multivariate regression analysis	The main barriers to adoption include cybersecurity and privacy and lack of trust in the government.	France, Germany, Italy, the United Kingdom, and the United States.
Nguyen et al. (39)	Extended TAM	Empirical, PLS-SEM, and fuzzy set/qualitative comparative analysis (fsQCA)	Risk perception, perceived usefulness, and health information orientation positively influence behavioral intention, which affects actual use.	United States
Hassandoust et al. (34)	Integrative situational PCT	Empirical, PLS-SEM	Risk beliefs, perceived individual and societal benefits to public health, privacy concerns, privacy protection initiatives (legal and technical protection), and technology features (anonymity and use of less sensitive data) are significant. In addition, there is an indirect relationship between trust in public health authorities and intention. Also, sex, education, media exposure, and past invasion of privacy did not have a significant relationship.	United State (US)
Meier et al. (41)	Privacy calculus perspective	Empirical, CB-SEM	Positive effects include perceived benefits and knowledge on actual app adoption, perceiving app benefits and usage intention, and trust with perceived benefits. However, those with negative effects include privacy concerns and usage, trust and privacy concerns, and privacy concerns with app usage.	Germany
Walrave et al. (35)	UTAUT	Empirical, CB-SEM	Performance expectancy, facilitating conditions, and social influence was significant, while effort expectancy was not. Moreover, individuals' innovativeness affects app use intention, whereas privacy concerns have a negative impact on intention.	Belgium
Duan and Deng (37)	UTAUT and PCT	Empirical, CB-SEM, and ANN	Effort expectancy, the perceived value of information disclosure, and social influence are significant. Moreover, perceived privacy risks and performance expectancy are indirectly significant. However, facilitating condition is insignificant.	Australia
Blom et al. (38)	NA	Empirical, descriptive statistics	Effectiveness of app-based contact tracing to contain the COVID-19 pandemic.	Germany
Dowthwaite et al. (40)	Extended TAM	Empirical, descriptive statistics	Differences in vulnerable populations' attitudes toward and trust in the app and compliance with self-isolation guidance were emphasized.	United Kingdom
Sharma et al. (36)	FT, DCT, PMT, TPB, and HCDT	Empirical; PLS-SEM	The relationship between the effectiveness of privacy policy and privacy concerns is negative, perceived vulnerability and privacy concerns is positive, expected personal and community-related outcomes of sharing information and attitudes is positive, privacy concerns and attitudes is negative, and attitude, subjective norms, and privacy self-efficacy on intention is positive.	Fiji

#### 2.1.2. Tawakkalna

The recent support by information technology has enable several countries implemented CTAs to restrict the spread of the COVID-19 virus. The Kingdom of Saudi Arabia (KSA) is among the countries that have harnessed technology innovation to manage the spread of the coronavirus. Thus, in the KSA's efforts and as part of its commitment to protect the health and safety of its citizens and residents from the risk of COVID-19 transmission, the government introduced an app called “Tawakkalna” to trace the health status of individuals and to permit them to enter public places. The app was developed by the Saudi Data and Artificial Intelligence Authority (SDAIA) to support government efforts to fight the coronavirus spread; this is along with other initiatives such as Tetamman, Tabaud, Sehha, and Mawid (42). In the nine months since Tawakkalna's inception, the app has accumulated over 17 million users (43). This result demonstrates the app's high reliability as one of the most successful and effective digital solutions.

As a result, the Tawakkalna app was built to support the digital issuance of movement approvals for private sector and government employees as well as individuals with emergency concerns. This is in collaboration with the Ministry of Health and other relevant authorities to limit the spread of the pandemic during the movement control order period. Individuals, as well as health, private, and security entities that are not primarily concerned with movement control, can use the app to automate all interactions between relevant parties, significantly reducing the economic, health, and social consequences of policies adopted to eradicate COVID-19 (6). Similarly, Tawakkalna assists managers of the crisis to monitor CTA users' health status. Moreover, the app provides other services, such as allowing individuals to report policy violations or any suspected cases through social responsibility and encouraging users to take precautions before leaving their homes (11). According to a survey conducted by (44), Tawakkalna has higher ratings for users' expectations in its key performance indicators (KPIs) than its counterparts, the Tetamman and Tabaud.

Furthermore, several studies in the literature have been conducted on the KSA's COVID-19 technology-supported management (42, 44, 45). Although, peak number of these studies focused on investigating the role of mobile applications in health or investigating the adoption factors without a link to adoption theory in the information systems literature. Hence, most of the existing work has not addressed the adoption factors based on the theoretical lens of information systems literature concerning technology adoption model, as well as the holistic picture of privacy risk concerns. Hence the focus of this study is to investigate the acceptance of the Tawakkalna app *via* the TAM, and the TTF and PCT theoretical models.

### 2.2. Theoretical Foundation

Technology adoption theoretical models are used to examine adoption behavior in the IS literature. The following sections discuss the models covered in this study. To be specific, the explanation of each model is then provided along with the reason why it is suitable for this study.

#### 2.2.1. Technology Acceptance Model (TAM)

The technology acceptance model (TAM), created by (46) and (47), comprises two fundamental factors, perceived ease of use (PEoU) and perceived usefulness (PU), and a third factor called attitude toward use (ATU). PU is defined as a person's belief that utilizing a system will improve his/her performance and PEoU as explaining a person's belief that using a system is effortless. Additionally, Davis et al. (48) emphasized that the actual usage of the system is influenced by the user's behavioral intention which is driven, in part, by the user's attitude toward the system's use and perceived usage (49). The TAM is employed in this study as it has been primarily acknowledged by academic researchers in the technology adoption literature (23, 50–52). Taherdoost (52) and Alwabel and Zeng (23) added that the TAM is arguably the most frequently quoted model in the field of technology acceptance. It has received strong empirical backing during past decades. Moreover, research by Turner et al. (53) suggested that, by using the TAM at the time of the release of a technology, the model should be able to forecast future usage of that technology. Similarly, the suitability of the TAM to predict new technology usage has been widely emphasized (51, 52). Research has suggested the use of acceptance theories, such as the TAM, in future CTA studies (8). Apart from being a widely compared model, the TAM, with some updates, has been utilized to assess the intention to use apps in various industries and contexts (54–56), including health app (57).

Benbasat and Barki (58) argued that many adoption contexts are required to understand diverse behavioral factors. Understanding specific behaviors would provide more significant recommendations for the design and practice of technology than just arguing for higher usefulness. CTA are still in their infancy, and just a few researchers have studied whether PU and PEOU perceptions continue to be sufficient to account for users' behavioral intents to use CTA. Although, CTA are sophisticated technologies that operate under various laws, settings, and features that vary based on social norms of a particular country (8, 36). No study has investigated the PU or PEOU from the Tawakkalna point of view. Therefore, this study employs the two independent variables of the TAM. Additionally, this work aims to close the gap in the literature on TAM and other technology adoption models by applying machine learning (ML) techniques to aid in developing a predictive technology acceptance model that has received little attention in the existing literature (23). To our knowledge, no study has used ML techniques such as artificial neural networks to predict PU and PEOU in CTA adoption. As a result, both PU and PEOU are required and appropriate for examination in this study.

#### 2.2.2. Privacy Calculus Theory (PCT)

According to privacy calculus theory (PCT), consumers make privacy-related decisions by weighing the benefits of any information disclosed against the risks of its exposure. Thus, this study focuses on the specific risks that influence users' acceptance of CTAs. The PCT has been employed to better understand consumers' assessment of the objectivity of disclosing private information (59–61). Furthermore, the PCT claims that consumers make privacy decisions by weighing the potential benefits against the potential risks posed by disclosing their personal information (62). Thus, the concept of privacy has a significant impact on how information is disclosed (63, 64), in the online context, privacy refers to the individual's awareness and control over the gathering and use of his/her personal data (65).

Prior research has established that a user's decision to download a new app is not always reasonable when the risks and rewards of information trading are evaluated. Instead, external pressures such as time restrictions, quick gratification, or optimistic bias impact on the decision, leading to acceptance of the advantages while ignoring the risks (66, 67). As consumers gain experience with mobile apps, they tend to focus on the benefits and downplay the potential hazards. Users may be unaware of the personal health data trade-off with a CTA when downloading the app. This research employs PCT to focus on the rational evaluation of risk-benefit calculations that users may undertake to determine whether to accept or reject this type of interaction. Using findings from earlier studies that employed PCT to explore electronic commerce (e-commerce) and mobile commerce (62, 68–70), and CTA adoption (36, 71), this research examines the critical risk and reward components of contact tracing, a hitherto understudied area. The hypothesis is that both perceived risks and advantages influence users' acceptance of Tawakkalna.

#### 2.2.3. Task-Technology Fit (TTF) Model

Over the past few decades, a significant amount of research has been conducted to understand behavioral intentions, including general use intention, actual usage, and continuous use of information systems (IS) *via* technology adoption models (46, 72, 73). Among several theoretical frameworks, the TTF model focuses on how newly developed technological solutions (IS products) fit a user's current tasks, hence boosting the user's performance (74–76). The assessment of how well the technologies incorporated in an IS-based product meet users' current tasks is the primary focus of the TTF model. The TTF model has been extensively applied in information system research and recently, it is frequently coupled with other models, such as the information system success model (77), social cognitive theory (SCT) (78), and the unified theory of technology acceptance and use (UTAUT) (79, 80). As with the TTF model, the use context established by an IT product is critical for its acceptability and use (81, 82).

The CTA was developed and introduced as a result of the integration of information technologies into health services. The amount of research conducted under the contact-tracing umbrella in the study of IT usage intentions has been substantial (10, 44, 45). The concept of mobility has been viewed as an explicit technological component within the TTF framework (79, 81, 83, 84). By utilizing a mobile app for a CTA, users' perceptions of the TTF model can be enhanced by the ability to perform certain activities anywhere and at any time (79, 83, 85), which, in turn, changes their behavioral intention toward the task. Mobile health apps used in emergencies can be classified as “general-purpose apps” or “built-for-disaster-purpose apps” (86, 87), with contact tracing falling under the latter category. Most CTAs have capabilities that allow interaction patterns between citizens and crisis management authorities during emergencies (88). The use of technology-mediated mobile apps is already ingrained in our societal structure. Therefore, in addition to understanding the mobile CTA, evaluating its technical characteristics, particularly its mobility, need to be evaluated. In light of the success reported in the literature of the TTF model on behavioral intentions toward mobile apps, this study applies the model *via* integration with TAM and PCT to better understand usage intentions toward the KSA's CTAs.

## 3. Model and Hypotheses Development

**Figure 2:**
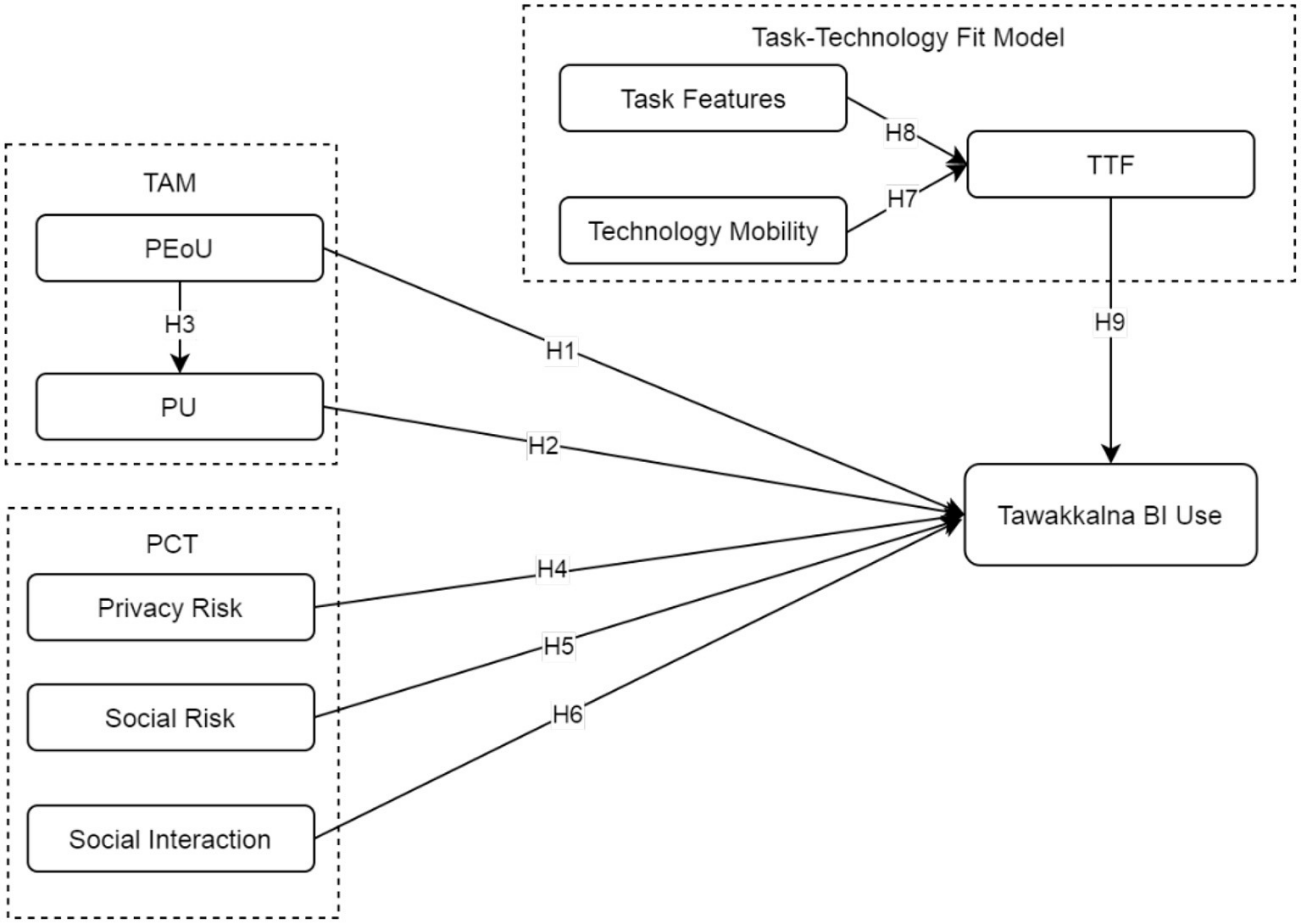
Research model for adoption of Tawakkalna contact-tracing app (CTA). BI, behavioral intention; PCT, privacy calculus theory; PEoU, perceived ease of use; PR, privacy risk; PU, perceived usefulness; SI, social interaction; SR, social risk; TAM, technology acceptance model; TF, task features; TM, technology mobility; TTF, task-technology fit.

Research on theory development has highlighted the need for integrating theories and models (34, 36, 37, 80, 89, 90). Based on the TAM, PCT, and the TTF model, this study develops and applies an integrated model to explain behavioral use intention toward the Tawakkalna COVID-19 tracing app, as shown in Figure 2. Behavioral intention (BI) has been described as the extent to which an individual has made deliberate decisions about whether to perform or not to perform a particular behavior or the probability of an individual's intention to engage in a specific behavior (91, 92). This study defines BI as the extent to which an individual will adopt or continue to use Tawakkalna in the future (71). Accordingly, the TTF model is influenced by both mobility and task characteristics. The TAM PEoU also has effects on perceived usefulness (PU). The BI is determined mainly by the TTF, PU, PEoU, perceived privacy risk, perceived social risk, and social interaction. The perceived social risk has both positive and negative effects on behavioral intention. The following section discusses the research model and its hypotheses in detail.

### 3.1. TAM Constructs

Earlier studies that used the TAM to examine public health IS apps discovered that PEoU and PU were the most prevalent and significant technology acceptance factors (93–95). Perceived usefulness is defined as the degree to which an individual believes that utilizing a system will reasonably improve his/her performance (46). Perceived ease of use is described as a person's belief that utilizing technology will involve minimal exertion (96). Similarly, according to the TAM, an individual's attitude toward a technological system is defined by these two variables: PU and PEoU. Moreover, Velicia-Martin et al. (10) recently investigated COVID-19 tracing app acceptance *via* the TAM's theoretical lens. The results of that study found that PEoU and PU were significant predictors of intention to use the CTAs. Hence, this study anticipates that the KSA population will embrace and use Tawakkalna due to the benefits associated with its use. As a result, the following hypotheses are formulated:

Hypothesis 1 (H1): Perceived ease of use has a positive and significant effect on behavioral intention of Tawakkalna.
Hypothesis 2 (H2): Perceived usefulness has a positive and significant effect on behavioral intention of Tawakkalna.
Hypothesis 3 (H3): Perceived ease of use positively and significantly affects perceived usefulness.

### 3.2. PCT Constructs

Internet-related perceived privacy risk refers to the extent to which internet users are concerned about how and to what extent an online entity collects and uses their personal information (97). This indicates a perceived disconnection between users' expectations and the reality of how their personal information will be managed (13). Numerous difficulties are arising in the context of COVID-19 crisis management that may impede users' acceptance of contact tracing. These difficulties cover the six main dimensions that shape internet privacy concerns: data collection, improper access, secondary usage, error, awareness, and control (98). The degree to which an individual is concerned about the amount of personal data that the internet app has gathered is characterized as part of data collection. Concerns about secondary data usage occur when an individual is worried that personal information might be used for another reason or shared with third parties without his/her consent. Improper access is when an individual is concerned about personal information being stolen or made available to unauthorized parties. Error focus refers to the accuracy of personal information and the techniques used to correct and maintain error-free personal data. Awareness refers to an individual's understanding of privacy terms and conditions. Control reflects the lack of adequate measures to control data collection and management.

Privacy concerns are critical in the COVID-19 tracing app context, as the app requires the acquisition of not only personal information but also location information, which many individuals regard as very sensitive due to the increased potential of information misuse (99–101). The sharing of personal information, such as health status and location details, is of particular concern to users of certain mobile apps (19). Additionally, several studies in the literature have already raised concerns about the impact of privacy issues in relation to CTAs (8, 10, 36, 71). As a result, it is reasonable to predict that Tawakkalna will be seen negatively by persons with a high level of perceived privacy concerns who are likely to view location tracking and information storage as a danger to their freedom and privacy. Thus, the following hypothesis is proposed:

Hypothesis 4 (H4): Perceived privacy risk has a negative and significant effect on behavioral intention of Tawakkalna.

Moreover, perceived risk has been characterized as the uncertainty, uneasiness, and worry felt by users when they cannot anticipate the repercussions of providing personal information online (102). This level of disclosure is enhanced in the mobile environment, as it enables the detection of a user's location, the time, and the presence of other connected users in the vicinity (62). Although perceived risk has been conceptualized as a multidimensional term encompassing financial, performance, physical, physiological, and social risk (13, 60). This study focuses on social risk as a particularly prominent component in the contact-tracing application.

In PCT, the readiness to disclose information is connected to a negative perception of risk and the perception of a favorable benefit. For example, by revealing location-based information or health status to authorities, individuals may benefit from physical movement (4, 16), which may increase the number of users inclined to use the contact-tracing app. On the other hand, location-based information is viewed as highly sensitive (16) by users who perceive this information as intrusive, who do not want their social activities to be threatened by authorities due to COVID-19 risk exposure, or who are fearful of being forced to self-isolate. Hence, the pandemic has significantly harmed individuals' capacity to have deep interpersonal relationships with other people and has severely impaired the extreme human need for contact, discouraging or containing any physical manifestation of attachment and connection (4, 103). The reason is that authorities are viewing reduced social interaction as the way to save millions of lives during the COVID-19 pandemic (104, 105). Moreover, the pandemic has displaced many people from their jobs and, crucially, from their social networks, forcing them to collaborate and maintain affiliations remotely and distantly (106). Thus, the following hypotheses are stated concerning social risk and social interaction benefits that could be derived from using a CTA.

Hypothesis 5 (H5): Perceived social risk has a negative and significant effect on behavioral intention of Tawakkalna.
Hypothesis 6 (H6): Perceived social interaction has a positive and significant effect on behavioral intention of Tawakkalna.

### 3.3. TTF Constructs

According to previous literature, the TTF framework has been modified to meet the nature of emergency response settings and, thus, it is integrated into the research model in its entirety. As previously stated, mobility is viewed as a distinguishing technological element of mobile contact tracing in this study. To be more precise, mobility is defined as the degree to which users perceive their ability to access and use mobile contact tracing at any time and from any location (79, 81, 82). This technological aspect is particularly beneficial for crisis and emergency responses, as an individual suffering a time-sensitive occurrence may immediately access the application (87, 107). As a result, the following hypothesis is formulated:

Hypothesis 7 (H7): Technology mobility is positively related to task-technology fit.

Moreover, in the TTF framework, task features/characteristics are typically interpreted as helpful behaviors that satisfy users' needs in using an information system (IS) (79, 82, 108). Typically, the Tawakkalna tracing app has a bundle of specific task characteristics that are reshaped to fit the study setting (84). Individuals can utilize the app to seek/share health information, engage with authorities, access COVID-19 vaccine, and report concerns (43, 88). Users can also execute various emergency response tasks within a single platform *via* their mobile at any time and from any location (87). As a result of these task features or characteristics, the following hypothesis is formulated:

Hypothesis 8 (H8): Task features or characteristics are positively related to task-technology fit.

Validation of H7 and H8 contributes to knowledge of the fit between mobility and emergency response tasks and, thus, are crucial to the research questions. According to (79), a strong match between task and technology promotes users' behavioral intentions, whereas a weak match has a negative effect on their behavioral intentions. Numerous studies in the literature on other mobile apps have demonstrated how consumers' perceived task-technology fit affects their adoption of a specific app (77, 79, 84, 85). For example, evidence on users' behavioral intentions regarding mobile learning demonstrates the effective implementation of the TTF model (108, 109). Similarly, an individual's perceived task-technology fit concerning emergency responses *via* mobile social media is a significant predictor of his/her behavioral intention toward mobile social media during emergencies (82). Thus, this study proposes that the TTF could impact on the success of behavioral intention toward Tawakkalna as the app is mobile-based and has learning, health, and social support. Hence, the following hypothesis is formulated:

Hypothesis 9 (H9): Task-technology fit positively and significantly affects behavioral intention of Tawakkalna.

## 4. Research Methodology

### 4.1. Data Collection Process

The targeted sample comprised residents of the KSA, including citizens and non-citizens, who were using the Tawakkalna app and were above 18 years old. Purposive sampling, a non-probability sampling strategy, was used in this study. It is defined as an approach in which targeted items meet particular requirements (110). Purposive sampling (judgement sampling) is the purposeful selection of a participant based on the participant's characteristics (111). It is a non-random strategy that does not require any underlying principle or a predetermined quantity of participants. The researcher uses this technique to survey a population that meets certain criteria for being viable for the study (110, 111). The researcher determines what information is required and then seeks out persons who can and will supply it based on their knowledge or experience (111). Purposive sampling improves the study's rigor and the reliability of the data and outcomes by better matching the sample to the research's goals and objectives (112). Moreover, the purposive sampling is an effective strategy for researching the early phases, when target participants have little or no expertise with the technology under inquiry (111, 112). Contact-tracking apps, notably Tawakkanla, are still in their infancy and consequently have a small user base (8). Additionally, this technique has been employed to study the adoption of existing IT (113). Thus, it is appropriate for this research.

In data gathering, purposive sampling can be used with several techniques (110). To recruit respondents, this study used the snowball sampling technique which follows purposive sampling (110, 112). Snowball sampling is the process of identifying participants “through referrals made among people who share or know of others who possess some characteristics that are of research interest” (114). This technique is suitable for this study due to the difficulty of obtaining a list of targeted users of Tawakkalna to ask them to describe their perceptions and experiences (110). A pre-test phase was conducted to validate and refine the questionnaire (115). Five information systems professors were asked to assess the survey questionnaire in order to confirm the questions and items' face validity. Minor revisions were made in response to their feedback. The survey was prepared on google form and the link was then sent by email and posted on two popular and highly used social network tools in the KSA, namely, WhatsApp and Twitter. The initial respondents were invited to forward the link to family members, friends, and coworkers, in order to encourage them to complete the questionnaire and share the URL with others (112). Two survey links were created to reveal the numbers recruited from the selected settings. In total, 1,080 clicks were accessed *via* the questionnaire link; 78% of responses were recruited from Twitter and WhatsApp, while 22% received an email invitation. Furthermore, responses that were incomplete (549), responses that were completed in less than the average time for survey completion (7 min) (193), and responses that indicated the respondent was under the age of 18 (30) were removed from the study during the data cleaning. Hence, the final sample consists of 309 records which were completed and ready for further evaluation. The data collection procedure was conducted in 2 weeks between March 1 and 15, 2021.

A sufficient sample size should be used to estimate a model's parameters (116). When using confirmatory factor analysis (CFA), (117) recommended that the sample size be greater than 300 in order to objectively and adequately accomplish the study. However, the work by Hair et al. (116) disputed this figure, stating that, for a minimum *R*^2^ (coefficient of determination) value of 0.25 with 5% error probability and 80% statistical power, the minimum sample size should be 45 for at least a maximum number of five arrows pointing to the dependent variable. Our sample is larger than those in similar SEM–ANN studies (118). Therefore, based on the criteria listed above and the statistical analysis method used in this study, the total sample size obtained (*N* = 309) is deemed adequate and sufficient for estimating the parameters of the model. As this study adopted partial least squares (PLS) and conducted CFA, the normality issue did not need to be considered for normally distributed variables (119, 120).

### 4.2. Item Development

The item creation phase is important to confirm the content validity of measurement items (120). The item measures utilized in this study were designed and tested previously in well-established research, with minimal alterations to match the purpose of this study (121). Hinkin (122) hinted that there is no strict rule governing the number of items that should be included in each construct. Although, it is critical to guarantee that each construct's domain is adequately sampled (123). Also, work by Gefen et al. (120) insisted that three indicators that are completely dependent on a single common factor can statistically detect the construct factor measurement model. Accordingly, most constructs in the current study were measured by at least three items (see Table A1).

Behavior intention (BI) was reflected by three items adopted from (71, 73). Perceived ease of use and usefulness scales were adopted from (47) and (10). Three items were created for task features (TF) (79, 82), while mobility (TM) (79, 81, 82) was measured using four items. Three items were employed for each of TTF (79, 82) and social interaction (SI) (103). Also, this study adapted four reflective indicators to measure each of privacy risk (PR) (13, 98) and social risk (SR) (13). A 7-point Likert scale was used as a multiple-item scale so respondents could rate their degree of agreement to record their responses. The scale ranged from “strongly agree” to “strongly disagree,” with “7” being strong agreement and “1” being strong disagreement (122). The questionnaire was written in English and translated into the Arabic language as responses were sought from all layers of Saudi citizens, providing them with the opportunity to participate.

### 4.3. Common Method Bias and Non-response Bias

The study findings may be vulnerable to common method bias (CMB), as survey data were self-reported, and behavior was not quantified as it was based on users' self-assessment (124). As a result, some methods were implemented to evaluate and mitigate the potential for CMB, as recommended by (125). To be specific (126), priori procedural remedies were incorporated. This method was utilized during the pre-test phase to refine the scale items and eliminate potential ambiguities, with multiple-choice questions periodically included to break up the pattern of questions rated using Likert scales. Moreover, to check for CMB, Harman's single-factor test was run. The result shows that the total variance was <50%, which is 40.72%, indicating that CMB was not an issue in this study (Table 2). Also, the path coefficients obtained from the structural model assessment had varying degrees of relevance (125).

**Table 2:** CMB total variance explained.

**Factor**	**Initial eigenvalues**			**Extraction sums of squared loadings**		
	**Total**	**% of variance**	**Cumulative %**	**Total**	**% of variance**	**Cumulative %**
1	13.302	41.568	41.568	13.032	40.724	40.724
2	3.327	10.398	51.966			
3	1.969	6.153	58.12			
4	1.492	4.663	62.783			
5	1.05	3.283	66.065			
6	0.981	3.065	69.13			
7	0.812	2.536	71.666			
8	0.766	2.395	74.062			
9	0.733	2.292	76.353			
10	0.696	2.174	78.527			

This study applied the non-response bias test (121). Thus, two subsamples were created based on the order in which respondents responded to the survey questionnaire. The first 75 early responders were divided into two groups, while the second 75 late respondents were divided into two groups. The two groups were compared using a two-tailed *t*-test with a 5% threshold of significance (127). There were no significant differences in the test outcomes between these two groups of responders. As a result, this study was not concerned with non-response bias.

### 4.4. Data Analysis Methods

#### 4.4.1. Structural Equation Modeling (SEM)

The study used a PLS-SEM technique to analyze the data. Two primary reasons prompted the researchers to select this technique: firstly, the PLS technique does not make a strict assumption about the normal distribution of data (128). Secondly, the PLS technique is preferable for composite analysis (129). Another reason for using the PLS-SEM technique was a dearth of established hypotheses for predicting behavioral intention toward the COVID-19 CTA, as well as the model's relative complexity, as it contained nine constructs. As such, the study evaluated the measurement model first by determining the constructs' reliability and validity, and then estimated the path coefficients and other structural model parameters through CFA based PLS-SEM approach, in accordance with available recommendations (129, 130). Therefore, SmartPLS 3 software was specifically employed throughout the SEM process.

#### 4.4.2. Artificial Neural Network (ANN)

The study re-examined the research model using an artificial neural network (ANN). The dual analysis was undertaken due to the benefits from both PLS-SEM and ANN strengths (22, 23, 131, 132). Thus, the ANN was used to determine the predictors of Tawakkalna use intention (133). The PLS-SEM technique is regularly used to examine and test causal relationships (116, 134). Moreover, Chan and Chong (134) and Teo et al. (135) reported that the ANN is utilized to identify complex linear and non-linear relationships. Although recent studies suggested that the ANN is not enough to handle a complex nonlinear relationship (136, 137). Nevertheless, the ANN approach give better results than conventional prediction methods (137) and is more precise than the usual regression strategy in terms of prediction (82, 138). As a result, the ANN approach has been utilized to examine the link between dependent and independent variables in IS research (22, 82, 132, 139, 140). Hence, this study employed ANN analysis to determine the factors that significantly influence Tawakkalna use intention.

## 5. Results

### 5.1. Descriptive Statistics

Table 3 shows the demographic profile of respondents. The sample mostly comprised male respondents (66.9%). Ages ranged from 18 to 41 (79.9%) and 21% were older than 41 years. The sample level of education shows that 90.4% have a Bachelor's degree or higher. With respect to mobile app usage, 78.3% of the sample used the apps between 1 and 7 h daily. This data is consistent with a previous survey report indicating that the average daily mobile usage time is between 5 and 6 h (141). Furthermore, according to the mean values, the number of responses falls between 4 and 7, which spans from 4.39 to 5.39. In addition, the standard deviation range is narrow, indicating that the values are near the mean. In addition, the data shows minor variations and deviations. Rumsey (142) defined the data concentration toward the mean as the respondents' agreement on the impact of factors on behavioral intention. Table 2 shows the results of the mean and standard deviation of the constructs.

**Table 3:** Demographic characteristics of sample.

**Category**	**Frequency**	**Percent**
**Gender**		
Male	207	66.9
Female	102	33.1
**Age (years)**		
<18	0	0
18–21	28	9.1
22–31	95	30.7
32–41	121	39.2
42-51	45	14.6
52–61	17	5.5
More than 61	3	1
**Education**		
Not educated	0	2.6
Secondary school	8	4.9
Diploma	15	58.3
Bachelor	180	14.6
Master	45	17.5
PhD	54	2.9
Others	9	
**Nationality**		
Saudi	226	73.1
Non-Saudi	83	26.9
**Mobile app day usage**		
<1 h	11	3.6
1–3 h	160	51.8
4–7 h	82	26.5
8–11 h	35	11.3
12–15 h	12	3.9
More than 15 h	7	2.3
Not specified	2	0.6

Moreover, to better confirm the normality distribution, the skewness and kurtosis tests were used to evaluate multivariate normality in the data (143). The skewness test verifies that the variable distribution is symmetrical by determining the most likely scenario (116). The ranges from −1.96 to +1.96 are used to determine if the data is normally distributed. However, if the sample size is more than 300, the thresholds for skewness and kurtosis are −2 to +2 and −7 to +7, respectively (144). The skewness and kurtosis results for this study are reported in Table 4. Hence, the data met the appropriate normality assumption based on the regression normality coefficient. As a result, the motivation to use path analysis using PLS is valid (116).

**Table 4:** Descriptive statistics: mean, standard deviations (S.D.) skewness, and kurtosis.

	**Mean**	**Std. deviation**	**Skewness**		**Kurtosis**	
	**Statistic**	**Statistic**	**Statistic**	**Std. error**	**Statistic**	**Std. error**
BI	5.175	0.957	−0.613	0.139	0.878	0.276
PU	5.188	1.039	−0.471	0.139	−0.132	0.276
PEoU	5.395	0.845	−0.513	0.139	0.311	0.276
PR	4.385	1.391	−0.476	0.139	−0.696	0.276
SI	5.272	0.999	−0.700	0.139	0.251	0.276
SR	5.232	0.961	−0.340	0.139	−0.384	0.276
TF	5.159	0.949	−0.293	0.139	−0.971	0.276
TM	5.215	1.037	−0.702	0.139	0.342	0.276
TTF	5.215	1.037	−0.702	0.139	0.342	0.276

### 5.2. Reliability and Validity

The reliability and validity of the study's constructs were determined by confirmatory factor analysis. To test the internal consistency reliability, the values for standard loadings, Cronbach's alpha, Rho_A, composite reliability (CR), and average variance extracted (AVE) were obtained for each construct, as summarized in Table 5. Most item loadings had values >0.7 and were statistically significant at *p* < 0.001 which is considered acceptable (119). In addition, each construct had an AVE value greater than the critical level of 0.5, indicating good convergent validity. Similarly, all constructs had values for CR, Rho_A, and Cronbach's alpha of more than 0.7, indicating good consistency dependability (115, 119, 129).

**Table 5:** Scale properties.

**Variable**	**Item**	**Standardized**	**Cronbach's α**	**Rho_A**	**CR**	**AVE**
		**loading**				
Behavioral Intention (BI)	BI1	0.901	0.829	0.836	0.898	0.746
	BI2	0.877				
	BI3	0.81				
Perceived Ease of Use (PEoU)	PEoU1	0.742	0.75	0.755	0.841	0.57
	PEoU2	0.788				
	PEoU3	0.781				
	PEoU4	0.707				
Perceived Usefulness (PU)	PU1	0.851	0.85	0.851	0.909	0.77
	PU2	0.903				
	PU3	0.878				
Privacy Risk (PR)	PR1	0.89	0.893	0.953	0.921	0.745
	PR2	0.932				
	PR3	0.782				
	PR4	0.841				
Social Interaction (SI)	SI1	0.825	0.79	0.791	0.877	0.705
	SI2	0.87				
	SI3	0.823				
Social Risk (SR)	SR1	0.762	0.827	0.838	0.886	0.662
	SR2	0.854				
	SR3	0.896				
	SR4	0.732				
Task Features (TF)	TF1	0.851	0.843	0.846	0.895	0.68
	TF2	0.818				
	TF3	0.819				
	TF4	0.809				
Technology Mobility (TM)	TM1	0.811	0.817	0.816	0.892	0.733
	TM2	0.901				
	TM3	0.854				
Task-Technology Fit (TTF)	TTF1	0.792	0.784	0.787	0.875	0.699
	TTF2	0.87				
	TTF3	0.845				

Furthermore, the discriminant validity of the variables was determined by comparing the square roots of AVE values and the inter-construct correlations for each construct, as recommended by (129), as presented in Table 6. Accordingly, the diagonal elements were significantly larger than the off-diagonal elements, indicating acceptable discriminant validity. Additionally, a heterotrait-monotrait (HTMT) criterion test was used to assess discriminant validity (129). The HTMT ratios were less than 1, correlating with the Fornell-Larcker criterion test results.

**Table 6:** Factor correlation coefficients and square roots of average variance extracted (AVE) values^*^.

	**BI**	**PEoU**	**PR**	**PU**	**SI**	**SR**	**TF**	**TM**	**TTF**
BI	0.864								
PEoU	0.617	0.755							
PR	−0.056	−0.073	0.863						
PU	0.673	0.546	−0.102	0.877					
SI	0.589	0.664	0.002	0.539	0.84				
SR	0.512	0.626	−0.059	0.519	0.747	0.814			
TF	0.682	0.609	−0.237	0.702	0.656	0.57	0.824		
TM	0.668	0.637	−0.075	0.707	0.626	0.558	0.726	0.856	
TTF	0.674	0.659	−0.071	0.632	0.692	0.633	0.762	0.757	0.836

* *Diagonal elements with bold font are the square roots of AVE values. BI, behavioral intention; PEoU, perceived ease of use; PR, privacy risk; PU, perceived usefulness; SI, social interaction; SR, social risk; TF, task features; TM, technology mobility; TTF, task-technology fit*.

### 5.3. Hypotheses Testing

**Figure 3:**
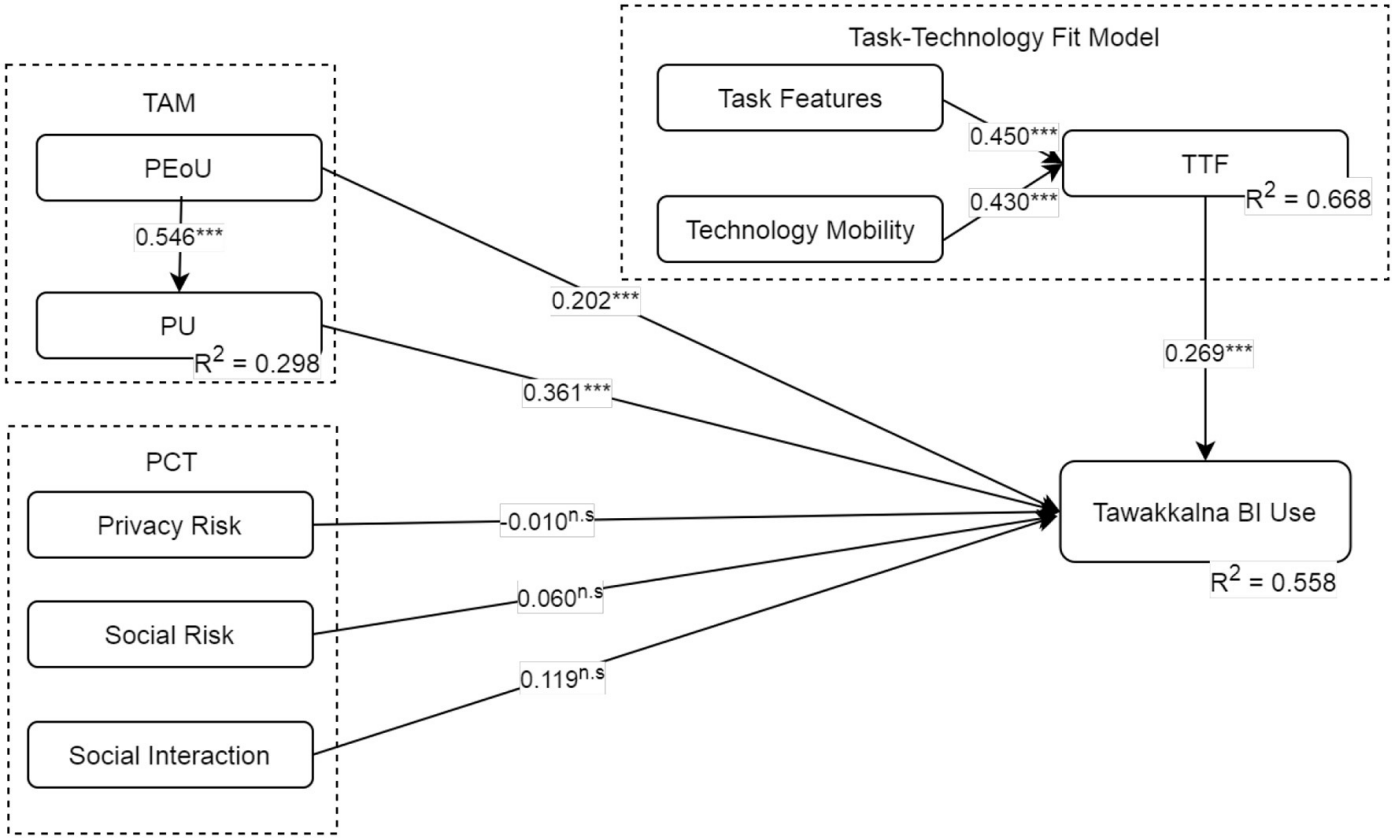
Test results of the structural model. n.s., non-significant; *p < 0.05; **p < 0.01; ***p < 0.001.

Various studies have recommended the bias-corrected and accelerated (BCa) bootstrap as the optimal method for discovering relationship effects (145–147). The study therefore ran a minimum of 5,000 bootstrap samples to provide a powerful method for assessing the model's hypothesized relationships (146). Figure 3 and Table 7 summarize and present the findings of the hypotheses' assessments. Except for the influence of variables derived from PCT's behavioral intention, all proposed relationships from the TAM and the TTF model were statistically supported, with 58.8% of the variance in the dependent variables explained by the model. Each of the TAM's variables was highly influential on BI, with the values of all relevant path coefficients exceeding 0.20, and PEoU explaining 29.8% of the variance of PU. As a result, hypotheses H1, H2, and H3 were supported. Regarding risk-related variables, the model produced some intriguing yet sensible conclusions. Both privacy and social risks were not endorsed; however, perceived social interaction had little effect (*B* = 0.119), but the hypothesis was rejected due to the *p*-value requirement. The result may show that rational decision-makers make sensible use of Tawakkalna to guide their movements to avoid COVID-19 high-risk areas. Among all variables, PU had the greatest influence on BI (*B* = 0.546, *t* − *value* = 16.713, *p* = 0.000). Additionally, the results associated with risks and benefits, specifically, social interaction associated with social risk, had little effect on BI (*B* = 0.119, *t* − *value* = 1.333, *p* = 0.182). As a result, H4, H5, and H6 were not approved. Also, when the indirect effects for the PEoU-PU-BI relationship were observed, the indirect effect of PU on use intention was *B* = 0.110, *t* − *value* = 2.730, *p* = 0.006, which was also significant.

**Table 7:** Summary of hypotheses.

**Hypothesis**		**Path coefficient (B)**	**SE**	**t-statistics**	***p*** **-values**	**Support**
H1	PEoU −> BI	0.202^**^	0.013	2.803	0.005	Yes
H2	PU −> BI	0.361^***^	0.012	5.564	0	Yes
H3	PEoU −> PU	0.546^***^	0.006	16.713	0	Yes
H4	PR −> BI	0.010^*n*.*s*.^	0.008	0.222	0.824	No
H5	SR −> BI	−0.060^*n*.*s*.^	0.014	0.747	0.455	No
H6	SI −> BI	0.119^*n*.*s*.^	0.016	1.333	0.182	No
H7	TM −> TTF	0.430^***^	0.009	8.372	0	Yes
H8	TF −> TTF	0.450^***^	0.009	9.04	0	Yes
H9	TTF −> BI	0.269^**^	0.014	3.451	0.001	Yes

*n.s., non-significant; SE, standard error;*

*p < 0.05;

** *p < 0.01;*

*** *p < 0.001*.

Moreover, the assessments of the task-technology fit hypothesis were all supported. Perceived technology mobility (TM) and task features (TF) both influenced perceived TTF at *B* = 0.430, *t* − *value* = 8.372, and *p* = 0.000; and *B* = 0.450, *t* − *value* = 9.040, and *p* = 0.000, respectively. As a result, the variables explained 68.8% of the variance in TTF. Therefore, H7 and H8 were also supported. Hence, task features (TF) had a slightly greater positive effect than perceived technology mobility (TM). Also, TTF had a favorable effect on behavioral intention of Tawakkalna (*B* = 0.269, *t* − *value* = 3.083, *p* = 0.001), supporting H8. Mobility and task characteristics had indirect impacts behavioral intention at *B* = 0.116, *t* − *value* = 3.083, and *p* = 0.002; and *B* = 0.121, *t* − *value* = 3.219, and *p* = 0.001, indicating support for indirect relationships.

### 5.4. Predictive Relevance and Effect Size

Additionally, the suggested model's predictive significance was tested using cross-validated redundancy (*Q*^2^), following the existing literature (129, 130, 132). All endogenous factors in Table 8 showed *Q*^2^ values >0, indicating that the model was predictive of PU, TTF, and BI. Additionally, (148) effect size (*f*^2^) was used to estimate each exogenous variable, as shown in Table 5. According to the magnitude of *f*^2^, 0.02, 0.15, and 0.35 correspond to small, medium, and large effects, respectively (130, 132). As indicated in Table 5, PeoU, PU, and TTF had a moderate to significant effect on BI, while the associated risk and benefits variables, namely, PR, SR, and SI, had a negligible impact on behavioral intention (BI). Also, TM and TF had a medium effect on TTF. Similarly, PEoU was a crucial antecedent of PU, having a medium impact. Notably, the associated *f*^2^ values that suggested non-significant effects were consistent with the path coefficient findings.

**Table 8:** *R*^2^, *Q*^2^ predictive relevance, and effect size (*f*^2^).

**Endogenous**	***R*** ^**2**^	***Q*** ^**2**^	***Exogenous***	***f*** ^**2**^
**variables**			**variables**	
BI	0.588	0.43	PEoU	0.045
			PU	0.176
			PR	0
			SR	0.003
			SI	0.012
			TTF	0.068
PU	0.298	0.151	PEoU	0.425
TTF	0.668	0.458	TF	0.288
			TM	0.263

### 5.5. Artificial Neural Network (ANN) Analysis Results

**Figure 4:**
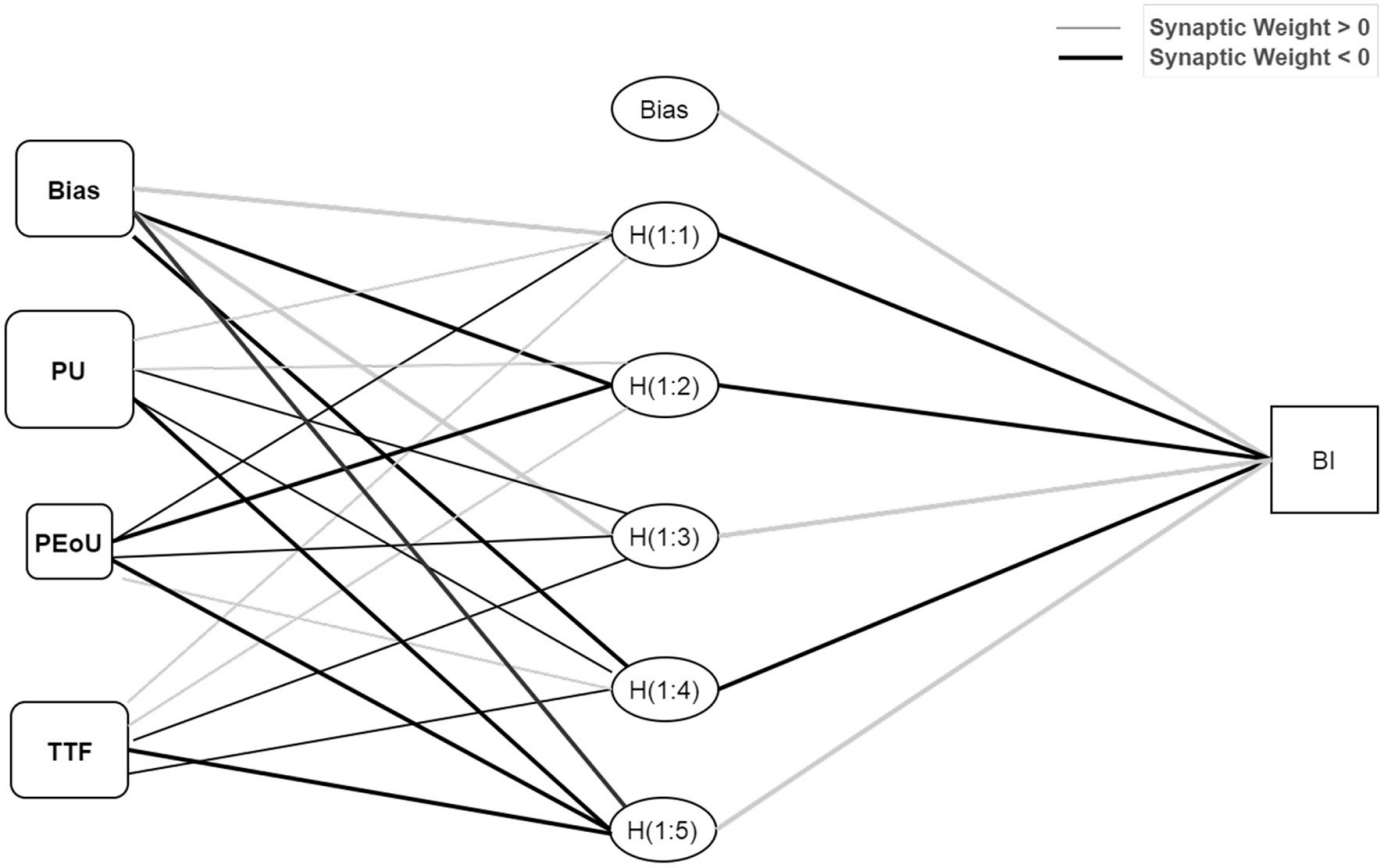
Artificial neural network (ANN) model. Hidden layer activation function: hyperbolic tangent; output layer activation function: sigmoid; input neurons: PU, perceived usefulness; PEoU, perceived ease of use; TTF, task-technology fit.

According to Chong (149), an ANN is a modeling tool capable of imitating human–neural systems and learning. Hence, the learning capabilities of ANNs allow them to be trained to improve their performance (135, 150). In this study, IBM SPSS Statistics (SPSS) v24 software was used to conduct the ANN analysis, following previously published and applied procedures (82, 131, 132, 151). This study identified the relative importance of exogenous elements to an endogenous variable using a multi-layer perceptron (MLP) artificial neural network (ANN) with a feed–forward back-propagation (FFBP) algorithm. Figure 4 depicts the ANN models developed using (152) neural networks drawing guidelines. To avoid overfitting, tenfold cross-validation was performed on the data set (resulting in 10 ANN models), with 70% of the data used for training and 30% for establishing the trained network's projected accuracy (also known as testing). Additionally, the algorithm generated a specified number of hidden neurons, with the hidden layer activated using the hyperbolic tangent activation function. In contrast, the output layers were activated using the sigmoid activation function. The root-mean-square error (RMSE) was determined for each network in the ANN model to determine the model's predictive accuracy, as indicated by numerous studies (82, 131, 135). As shown in Table 9, the ANN model has a mean RMSE of 0.545 for training data and 0.553 for testing data, showing the model's prediction capacity. Apart from implying a level of predictive accuracy, a lower RMSE value indicates a more precise fit and forecast of the data. Additionally, the relevance of external variables was assessed by the number of hidden neurons in an ANN model with non-zero synaptic weights.

**Table 9:** Root-mean-square error (RMSE) values for training and testing.

**Network**	**Training**	**Testing**
1	0.536	0.553
2	0.531	0.563
3	0.564	0.551
4	0.543	0.552
5	0.549	0.551
6	0.539	0.559
7	0.544	0.549
8	0.545	0.544
9	0.55	0.555
10	0.551	0.547
Mean	0.545	0.553
SD	0.009	0.006

*Network and input neurons: perceived usefulness, perceived ease of use, and task-technology fit; SD, standard deviation*.

After establishing the ANN model's anticipated accuracy and predictive relevance, a sensitivity analysis was conducted to statistically evaluate the exogenous variables' predictive capability concerning endogenous components (135, 153). The relative importance of each exogenous variable was determined, and the normalized relative value was computed, as indicated in Table 10. The exogenous variables were then ranked according to their normalized relative relevance and influence strength. Interestingly, when the three variables were examined on the ANN models, perceived usefulness was the strongest predictor of Tawakkalna behavioral intention, with 100% normalized relative importance. Moreover, the result of the relative importance of TTF (66.45%) and perceived ease of use (35.06%) were significant predictors of behavioral intention, in that order. Perceived usefulness was conclusively the strongest predictor of behavioral intention, whereas perceived ease of use was the weakest.

**Table 10:** Sensitivity analysis.

**Network**	**PU**	**PEoU**	**TTF**
1	0.501	0.165	0.334
2	0.465	0.188	0.348
3	0.558	0.138	0.304
4	0.452	0.181	0.367
5	0.54	0.173	0.287
6	0.477	0.202	0.321
7	0.448	0.165	0.387
8	0.465	0.159	0.376
9	0.567	0.175	0.258
10	0.524	0.191	0.284
Average RI	0.4997	0.1737	0.3266
Normalized RI (%)	100	35.06	66.45

*Input neurons: perceived usefulness, perceived ease of use, and task-technology fit; RI, relative importance; PU, perceived usefulness; PEoU, perceived ease of use; TTF, task-technology fit*.

## 6. Discussion

This study's primary purpose was to determine the key factors influencing users' behavioral intention of a CTA. In this research, an integrated model was developed that combined three perspectives: firstly, behavioral intention is driven by a user's perceived ease of use and usefulness. Secondly, BI is driven by perceived risk and corresponding benefits, classified as privacy risk, social risk, and social interaction. Thirdly, BI is influenced by the fit between technology mobility and task features. Accordingly, the survey data supported six hypotheses in the proposed model and revealed some intriguing findings.

The TAM is considered a valuable model for predicting the behavioral intention of a new app that tracks individuals who have been exposed to a positive COVID-19 case, hence breaking the infection chain (10). Therefore, the hypotheses for the TAM constructs, perceived ease of use and perceived usefulness, were supported by the findings. Interestingly, the variable with the largest significant impact was perceived usefulness. As these hypotheses were derived from the original TAM, the theoretical TAM can be used to examine the adoption of apps to prevent and mitigate the COVID-19 pandemic's effects. These findings corroborate existing studies that employed the extended TAM, testing both perceived ease of use and perceived usefulness as significant factors of use intention (10, 154).

The variables derived from PCT were not found to be significant in the proposed model. Specifically, privacy risk was not significant. Hence, the privacy risk concern was not found to effect the intention to use the tracing app. Thus, H4 was not supported. This suggests that privacy risk concerns about the app, such as the associated risk (from utilizing private information), had no adverse effect on the behavioral intention of the tracing app. This finding was supported by several studies in the literature (10, 155). Our finding also is in the line with previous studies that found insignificant influence between behavior adoption and privacy risks and concerns. These studies include privacy risk on intentions toward IoT health services (156), privacy concerns on Mobile health technologies (157) and CTAs (71).The privacy risk hypothesis was obviously in relation to health concerns. As simply stated by (158), individuals are more concerned about their health, enhancing their readiness to use CTAs developed for this purpose, even at the risk of jeopardizing their personal privacy (19). Li et al. (159) added that the perceptions of the benefits of CTAs are stronger predictors of behavioral intentions than the perceptions regarding security and privacy risk.

Likewise, in relation to the effect on BI, the analysis revealed that social risk and social interaction outcomes were not significant. Thus, findings revealed that the effects of risks, as proposed in H5 and H6, were not significant. The study's findings did not support those in prior research about the social impact of COVID-19 that emphasized the effect of the coronavirus on relationships among the public despite the human need for social contact (4, 106). Nevertheless, the finding could be consistent as the restriction of movement due to pandemic-imposed physical distancing creates a distressing awareness that one's wellbeing depends on others. This is true when comparing the benefits of CTAs to protecting oneself from getting COVID-19 with the combined risk of losing privacy, security, and the social risk (19, 159).

Similarly, privacy and perceived risks were found to have no relationship with behavioral intention. This could be a result of the variable's narrow assessment, which was limited to social and privacy risk perceptions. However, incorporating additional privacy concerns, such as uncertainty avoidance, perceived severity, and vulnerability (36), as well as risk perceptions, such as uncertainty, time, performance, and psychological loss, may provide a more full picture of these risk variables, which may bolster their perceived importance. As a result, researchers are urged to conduct comprehensive studies on risk perceptions associated with the acceptance of CTAs such as Tawakkalna.

Furthermore, in assessing the TTF predictors, the findings indicated that users' perceptions of the mobility of Tawakkalna were ideally aligned with their task characteristics, which influenced their behavioral intention of the tracing app. Mobility and task features accounted for 68.8% of the variance in the TTF in the model which had a more significant effect. The findings supported the earlier IS research regarding the influences of TTF on use intention (77, 82, 84, 160). This study emphasized the critical role of TTF in meeting the users' CTA needs. Also, the findings revealed that perceived usefulness was the most important direct antecedent of BI, followed by TTF. Thus, this research offers significant knowledge about the relative value of TTF as a predictor of a CTA's behavioral intention.

It is worth mentioning that the use intention explained a 58.8% variance in the proposed model. Compared to other variables, TTF had a higher predictive *R*^2^ value at 66.8%, with perceived usefulness explaining only 29.8%. Also, the *Q*^2^ assessment revealed that the predictive accuracy of TTF was slightly higher than the use intention, at 0.458 and 0.430, respectively. On the other hand, perceived usefulness had the lowest *Q*^2^ value (0.151). Thus, the results for the *R*^2^ values were aligned with the predictive potential of the model. Moreover, the ANN analysis results were in line with the SEM path coefficient outcomes, which ranked the variables according to their impact on the model. Perceived usefulness was the strongest factor, with TTF the second strongest.

### 6.1. The Optimality of the Proposed Approach

The information system literature about the technology adoption model primarily predicts behavioral intention, usage intention, or adoption intention as the dependent variable. Most of the studies investigate this variable predictive power through PLS-SEM (10, 34, 39) or CB-SEM (35–37, 41). One of the primary criteria used to examine the model's predictive capacity is the coefficient of determination (*R*^2^). Since this study adopted the PLS-SEM approach, the *R*^2^ values of existing studies are compared with the current studies. This is to show the novelty and optimality of the proposed approach. Hence, the comparative analysis revealed an exciting result. Firstly, studiess by Velicia-Martin et al. (10) and Hassandoust et al. (34) have higher *R*^2^ values than the proposed approach. However, these studies adopted a single theory, while our approach integrated three theories (TAM, PCT, TTF). Secondly, since existing studies based on PLS-SEM have not integrated multiple theories, this study compared the result of the proposed approach with the work by (36), which is based on CB-SEM and incorporated multiple theories (FT, DCT, PMT, TPB, and HCDT). Remarkably, the *R*^2^ of the proposed approach is slightly higher than the CB-SEM approach. Thirdly, the proposed approach is the first to apply two-stage SEM–ANN analysis among the existing studies based on the PLS-SEM approach. Moreover, similar studies in the context of middle-east countries, specifically KSA, are lacking. Thus, this study has addressed this gap to allow policymakers to take systematic steps to address these issues and enhance CTA acceptance. Table 11 presents the comparative analysis of the previous studies and the proposed approach.

**Table 11:** Comparison of proposed approach with existing studies.

**Category**	**Single theory**			**Multiple theories**	
Ref	(39)	(34)	(10)	(36)	Proposed model
Country	US	US	Spain	Fiji	KSA
Theoretical model	Extended TAM	PCT	Extended TAM	FT, DCT, PMT, TPB, and HCDT	TAM, PCT, TTF
Method	PLS-SEM and fsQCA	PLS-SEM	PLS-SEM	CB-SEM	PLS-SEM-ANN
Dependent Variable	App use	Intention to install CTA	Behavioral intention to use	Adoption intention	Behavioral intention
*R*^2^ (%)	44	75	77	51	56

## 7. Contributions and Implications

### 7.1. Theoretical Contributions

Numerous theoretical contributions are made by this study and its findings. The study examines the significant factors in the use of CTAs to determine users' behavioral intention through the lens of the TAM, and the PCT and TTF theoretical models. Despite the advent of health apps, particularly tracking apps, and their use to restrict the transmission of the COVID-19 virus, an information system perspective on the interaction between adoption determinants remains necessary.

Firstly, the TAM was used to understand the predictive potential of behavioral intention (10, 54, 161). The TAM's key constructs were then incorporated into Tawakkalna, a new technology, to reveal the key factors of users' behavioral intention. The key variables of the TAM contributed significantly to the model and improved the investigation's outcomes. The TAM variables thus explain why IS and geolocation technology have been adopted to prevent or reduce coronavirus transmission.

Secondly, through PCT, it was revealed that the public might be persuaded to embrace the app and to sacrifice their privacy and social concerns. Still, complications could arise due to the loss of privacy, higher-level notifications on devices, or the software's incompatibility in other countries (10). Accordingly, one of this study's key focuses is the negative effect of privacy on behavioral intentions. Several studies have focused on users' privacy on tracing apps, the measures to protect people's privacy (162, 163), or individuals' concerns regarding privacy issues (10). This study reveals that when it comes to health issues, individuals are unconcerned with privacy risk. These outcomes support the findings by (10) regarding the insignificant impact of privacy. Additionally, the positively or negatively evaluated social risk and social interaction have not yielded positive results. This does not support assertions by Schleicher (4) and Settersten et al. (106) regarding losing relationships among the public.

Thirdly, while examining the TTF variables, the data reveal that users' perceptions of Tawakkalna's mobility are optimally connected with their task characteristics which influence their behavioral intention. Mobility and task characteristics have indicated a more significant effect on technology fit (77, 82, 84). Hence, this study emphasizes the vital importance of the TTF in addressing the satisfaction of users' needs by mobile CTAs. To our knowledge, this is the first time the TTF model has been used to investigate the adoption of CTA. Therefore, the study offers a theoretical contribution that advances the understanding of the effectiveness of TTF on behavioral usage.

Furthermore, IT adoption studies, using a hybrid measurement analysis, are lacking. Studies have suggested that more research should be undertaken that combines ML tools with structural equation modeling (SEM) (22, 23, 82, 132, 149). Hence, this study establishes the proportional importance of critical characteristics that precede the behavioral intention of Tawakkalna by using the two-stage SEM–ANN analysis to address this gap by ranking these antecedents. The core factor of behavioral intention, namely, PU, helps users to adopt the app, while TTF is ranked second. These factors are shown to be conceptually and practically relevant, and this work contributes to the body of knowledge by directly measuring their relative importance utilizing a two-stage analysis.

This is the first empirical study to examine CTAs from a KSA perspective. Previously, CTA investigations have taken place in various countries, such as the UK (164), Germany (165), USA (159), Ireland (71), and Fiji (36). The differences in culture, infrastructure, legislation, and economies could impact on individuals' decision making (36). Research on theory development has emphasized the importance of measuring theories and models in the contexts of different countries (80). The findings of this study therefore contribute in this regard.

### 7.2. Practical Implications

COVID-19's spread has resulted in a pandemic, infecting millions of people and killing thousands. The use of notable measures by numerous countries has contained the spread of the coronavirus, enabling healthcare centers to care for the sick. However, this pandemic's constraints have severely affected the world economy. Moreover, stakeholders are demanding that movement control mechanisms should be enforced to control the spread. Health officials must be able to discover positive cases early and follow their prospective contacts. As a result, numerous technology-based tracking methods have been proposed, including mobile apps (1).

Even though the balance between health and the economy must favor the former, the economy has been severely harmed. Thus, tracking technology should be integrated into people's movements in order to protect population safety and facilitate the isolation of new positive cases and their associated contacts. This type of technology has been beneficial in countries such as Malaysia, South Korea, and Japan that were able to rapidly and safely decrease COVID-19's spread, reviving their economies through using geolocation-based apps (166, 167). The study's findings indicate that users' behavioral intentions (BI) are decided by the app's perceived usefulness (PU), perceived ease of use (PEoU), and task-technology fit (TTF). Concern about possible risks such as privacy risk, social risk, and associated benefits were not viewed as equally important by respondents. The choice of health was evident among respondents when they were confronted with choices, such as privacy and social risks.

Correspondingly, the knowledge obtained through this study will be beneficial to app developers who develop geolocation apps and to governments that decide on their use and the associated loss of privacy. Many discussions have been held concerning the loss of privacy rights, especially in liberal countries such as France, the UK, and the USA (8). Governments may find this study incredibly beneficial as they learn that users are particularly concerned about becoming infected or infecting their family members, with this concern significantly impacting on their decision to use the app. The user's intention is also influenced by his/her perception of vulnerability to COVID-19 coronavirus infection. Therefore, both anxiety and a sense of vulnerability, when combined with the perception of a high risk of COVID-19 infection, have a noticeable effect on behavioral intention. These findings corroborate the findings of recent studies (10, 166).

## 8. Future Work and Limitations

The study acknowledged few limitations. Firstly, respondents are from the KSA, demonstrating that our findings could only be applied to one geographical location. According to Li et al. (159), worries related to public health activate the behavioral immune system. This evolutionary adaptive process is culture-independent and, hence, the study's outcomes should be culture-independent. Therefore, the significant cross-cultural disparities in privacy concerns and potential users who did not use the CTA because of privacy concerns merit additional research. As mentioned earlier, certain cultures, such as those in China, Malaysia, and Japan may be more receptive to the government's acquisition and surveillance of personal data than cultures in more liberal countries.

The pandemic's long-term impact is unknown, although it is guaranteed to last longer than anticipated (4). However, one of the pandemic's most significant and immediate repercussions has been the way it has shattered the relationships among the public. While social participation may influence individuals' ability to adapt during a pandemic, physical separation measures also reveal and alter the character of the relationships among the public. Hence, this study suggests that this social issue is cause for concern. Important theoretical contributions might be made by explaining the mechanisms underlying these user's behaviors, particularly when weighing the risks and advantages of adopting CTAs.

Additionally, age has been demonstrated to be a moderator in numerous research published in the literature (80, 89) and CTA studies (159). The implication is that significant effects could potentially be tempered by age. However, this was not the study's objective, with no comparisons made between age groups. Consequently, future research could examine the acknowledged moderating effect of age. Finally, our findings may apply to other m-health technologies besides CTAs, such as electronic health records and wearables, although we lack the data to conclusively make this claim. As a result, additional research should be conducted to determine whether age acts as a moderator of the significance of the impact and to determine whether prominent disease concerns influence the adoption of other m-health technologies.

Furthermore, certain methodological limitations apply to the study. This includes the point that the data were collected through the snowball sampling approach. Hence, the sample is neither random nor fully representative of the population; this could result in a skewed sample of respondents, raising the issue of socially desired responses. Future research could use other data collection techniques. Moreover, the proposed model does not cover some factors such as government support, facilitating conditions, and social influence. Examining these factors could enhance our understanding of CTA adoption. Therefore, future studies may consider investigating their effect on behavioral intention to adopt Twakkalna and other CTAs. Also, future studies could investigate the impact of trust, as widely reported in the technology adoption literature (22) and as one of the key variables in the extended TAM (TAM2) (168).

## 9. Conclusion

The search for interventions to quickly and effectively control COVID-19's spread, which has endangered humans despite the increasing efforts committed to managing the pandemic, motivated the researchers to undertake this study. A mobile contact-tracing app is introduced to help KSA citizens to take precautions and to enable health authorities track potential positive cases. The app has various functions, including controlling the spread of the virus without impeding citizens' movement and consequential economic loss. As a result, this study examines the various factors that can affect the app's acceptability by the general population. Based upon the TAM, and PCT and TTF theoretical models, which are commonly used theories regarding behavioral intention, the study investigated the antecedents of Tawakkalna contact tracing as conducted in the Kingdom of Saudi Arabia. Of the nine hypotheses, the six corresponding to TAM and the TTF model were supported. Specifically, PU and PEoU were found to be significant predictors of behavioral intention. Also, the findings demonstrated that three constructs derived from PCT, namely, privacy risk, social risk, and social interaction, were not significant determinants of behavioral intention. Furthermore, users' perceived mobility and task features to use Tawakkalna matched perfectly, explaining 66.8% of the variation of task-technology fit. These conclusions are based on a two-stage SEM–ANN analysis coupled with different testing methodologies. In conclusion, the findings may shed new light on the overall role of contact-tracing apps.

## Data Availability Statement

The original contributions presented in the study are included in the article/supplementary material, further inquiries can be directed to the corresponding author/s.

## Author Contributions

All authors listed have made a substantial, direct, and intellectual contribution to the work and approved it for publication.

## Conflict of Interest

The authors declare that the research was conducted in the absence of any commercial or financial relationships that could be construed as a potential conflict of interest.

## Publisher's Note

All claims expressed in this article are solely those of the authors and do not necessarily represent those of their affiliated organizations, or those of the publisher, the editors and the reviewers. Any product that may be evaluated in this article, or claim that may be made by its manufacturer, is not guaranteed or endorsed by the publisher.

## Appendix

**Table A1:** Research items.

**Constructs**	**Code**	**Items**	**References**
Mobility	TM1	Using Tawakkalna mobile contact-tracing app is independent of time.	(79, 81, 82)
	TM2	Using Tawakkalna is independent of place.	
	TM3	Using Tawakkalna is convenient because the phone is usually with me.	
Task features	TF1	I need to accomplish some tasks at any time, e.g., scanning QR code to enter places.	(79, 82)
	TF2	I need to accomplish some tasks anywhere, e.g., scanning QR code to enter places.	
	TF3	I have some tasks which need to be completed immediately, e.g., scanning QR code to enter places or reporting violators.	
	TF4	I have some tasks which need to be completed importantly, e.g., registering an appointment for vaccination.	
Task–technology fit	TTF1	In helping to complete my tasks, the functions of Tawakkalna mobile contact-tracing app are enough.	(79, 82)
	TTF2	In helping to complete my tasks, the functions of Tawakkalna are appropriate.	
	TTF3	In general, the functions of Tawakkalna fully meet my needs, either when I am in an emergency or when I am not.	
Perceived usefulness	PU1	Using the Tawakkalna app would make me feel better about myself.	(10, 46)
	PU2	By using the Tawakkalna app, I would hope to be helping society.	
	PU3	The use of Tawakkalna app would increase my peace of mind.	
Perceived ease of use	PEoU1	The purpose of Tawakkalna is clear and understandable.	(10)
	PEoU2	I think that learning to use Tawakkalna would be very easy for me.	
	PEoU3	With Tawakkalna, it would be easy for me to avoid the contagion of COVID-19.	
	PEoU4	I would find it useful to have Tawakkalna to tell me how to avoid people who have COVID-19.	
Behavioral intention	BI1	I intend to continue using Tawakkalna in the future.	(71, 73)
	BI2	I will try to use Tawakkalna in my daily life.	
	BI3	I intend to continue to use Tawakkalna frequently.	
Privacy risk	PR1	It bothers me when health authorities track my location through my Tawakkalna mobile contact-tracing app.	(13, 98)
	PR2	I am concerned that health authorities are collecting too much location information about me through my Tawakkalna mobile contact-tracing app.	
	PR3	It bothers me if health authorities collect my mobile location and I cannot alter the location settings.	
	PR4	It bothers me when I do not have control over how my mobile location is used by health authorities.	
Social risk	SR1	I am concerned that my movement may be restricted if I do not use Tawakkalna.	(13)
	SR2	I am concerned that I may not be allowed to visit places without Tawakkalna installed on my phone.	
	SR3	I am concerned that my family and friends will not allow me to visit them if I am not using Tawakkalna.	
	SR4	I am concerned that if Tawakkalna indicates that I am a suspected case, I will be forced to self-isolate.	
Social interaction	SI1	By using Tawakkalna, I will be allowed to move freely, without any restrictions.	(103)
	SI2	By using Tawakkalna, I will visit places, anywhere and at any time.	
	SI3	By using Tawakkalna, my family and friends will allow me to visit them.	
